# Forecasting the Endemic/Epidemic Transition in COVID-19 in Some Countries: Influence of the Vaccination

**DOI:** 10.3390/diseases11040135

**Published:** 2023-10-03

**Authors:** Jules Waku, Kayode Oshinubi, Umar Muhammad Adam, Jacques Demongeot

**Affiliations:** 1 IRD UMI 209 UMMISCO and LIRIMA, University of Yaounde I, Yaounde P.O. Box 337, Cameroon; jules.waku@gmail.com; 2 AGEIS Laboratory, UGA, 38700 La Tronche, France; oshinubik@gmail.com; 3 Department of Mathematics, Federal University, Dutse 720222, Nigeria; umargdrone@gmail.com

**Keywords:** contagious disease, endemic phase, epidemic phase, endemic/epidemic transition forecasting, COVID-19 wave prediction

## Abstract

Objective: The objective of this article is to develop a robust method for forecasting the transition from endemic to epidemic phases in contagious diseases using COVID-19 as a case study. Methods: Seven indicators are proposed for detecting the endemic/epidemic transition: variation coefficient, entropy, dominant/subdominant spectral ratio, skewness, kurtosis, dispersion index and normality index. Then, principal component analysis (PCA) offers a score built from the seven proposed indicators as the first PCA component, and its forecasting performance is estimated from its ability to predict the entrance in the epidemic exponential growth phase. Results: This score is applied to the retro-prediction of endemic/epidemic transitions of COVID-19 outbreak in seven various countries for which the first PCA component has a good predicting power. Conclusion: This research offers a valuable tool for early epidemic detection, aiding in effective public health responses.

## 1. Introduction

### 1.1. Problem Statement

This study aims to develop a novel method for predicting transitions between endemic and epidemic phases in contagious diseases, with a specific focus on COVID-19 dynamics. To predict qualitative changes in the dynamics of a contagious disease, it is not enough to have a good mathematical model considering the mechanisms of contagion. It is also necessary, from the observed data, for example, new daily cases, to be able to predict the occurrence of a new epidemic wave from a stationary endemic situation as defined by D. Bernoulli in 1766 [1,2].

Such an objective requires the use of specific predictive statistical tools. To find a reliable method of prediction of the frontiers between different stationary and non-stationary phases of a time series is a challenging problem. This objective is close to that of the stationarity rupture tests studied for about forty years by statisticians. Indeed, since the seminal work by J. Deshayes and D. Picard on the stationarity rupture in time series [3,4], many works have dealt with stationarity breaking [5,6,7,8,9,10], the most recent using the concept of functional statistics, which considers observed curves of incidence or mortality as functions to be estimated in parametrized sets of functions [11,12,13,14,15,16,17].

### 1.2. Significance of the Research

**Figure 1:**
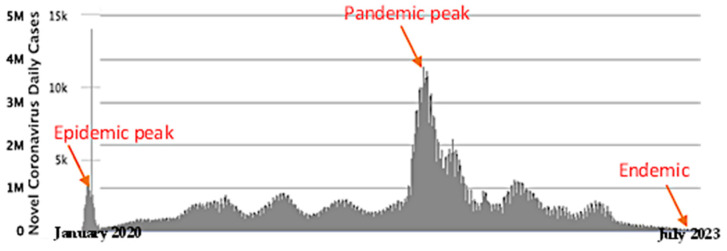
COVID-19 outbreak. Daily new cases in the world (after [35,36]).

There are very few relevant articles that have considered endemic/epidemic transition forecast, but our approach is different from other approaches in the literature. We intend to fill the gap; in addition, we seek to present a new method able to forecast the endemic/epidemic transition, taking as example the COVID-19 outbreak. In literature [18], some authors exploited the knowledge on the past epidemics, namely at the level of the endemic/epidemic transitions (see Figure 1), for making predictions on their occurrence during the COVID-19 pandemic [18]. In [19], the authors remarked that during the transition to the endemic phase, vaccination rates have lagged and that developed countries needed to boost vaccination rates globally.

The constraint of stationarity is crucial as many forecasting models of time series rely on stationary for performing an easy modeling and obtaining reliable results. The main characteristic parameters of the empirical distribution of the random variables of a stationary time series (moments, coefficient of variation, entropy, etc.) remain constant, the randomness coming often from an additive Gaussian noise. In the event of a break in stationarity, there may be a sudden transition with a sudden change in the values of these parameters and the appearance of a non-constant trend. The problem of the existence of this transition arises with acuity in the case of contagious diseases, which alternate stationary endemic periods and epidemic peaks with an exponential initial trend, which must be predicted to prevent the spread of the disease does not give rise to a pandemic.

### 1.3. Prediction Approaches in the Literature

The prediction of epidemics is one of the major objectives of the mathematical modeling of the spread of infectious diseases. It can be achieved by the spatiotemporal continuation of the solutions of the partial differential equations of the chosen continuous model realized through the extrapolation of a discrete statistical description of the evolution of the observed variables. The difficulty of predicting the evolution of a pandemic lies in the adaptive capacities of the infectious agent and the infected and transmitting host. On the one hand, the genetic mutations of the infectious agent and its contagious power and pathogenic dangerousness develop highly infectious and low pathogenic variants, often signaling the natural end of a pandemic. On the other hand, the permanent adaptation strategy of the individual and collective host defenses makes it possible to anticipate the effects of changes in the agent’s infectious strategy. In both cases, modeling the dynamics of mutation and prevention is essential to predict and act in near real-time on the evolution of a pandemic. We refer to [18,19,20,21,22,23,24,25,26,27,28,29,30,31,32] for more results and references on the topic of forecasting the contagious diseases.

### 1.4. Methodology and Approach

In this article, we offer a method to estimate the breakdown of endemic stationarity based on seven parameters whose isolated or joint predictive power is analyzed. These parameters are the coefficient of variation and the entropy of the empirical measure calculated in a moving window, as well as the ratio between the modules of the dominant and subdominant eigenvalues of the nonstationary transition matrix, the third and fourth standardized moments of the empirical distribution (called respectively skewness and kurtosis), and eventually diversity and normality indices, quantifying the distance of the empirical distribution to respectively the uniform and the normal distribution. 

An epidemic corresponds to an unexpected increase in the number of disease cases in a specific location. Yellow fever, smallpox, measles, and polio are prime examples of epidemics. An epidemic disease does not necessarily have to be contagious. The rapid increase in obesity is also considered as an epidemic: worldwide obesity has indeed nearly tripled between 1975 and 2016 and has been considered by WHO as a pandemic since 1997 [33]. 

A pandemic is characterized by exponential growth of the disease, when it concerns a continent of the entire world. This means the growth rate skyrockets, and each day cases grow in number more rapidly than the day prior. In being declared a pandemic, the virus has nothing to do with virology, population immunity, or disease severity. It means a virus covers a wide area, affecting several countries and populations [34]. A common example we experienced recently is the COVID-19 worldwide pandemic with a high contagiousness of the virus.

An endemic disease designates a disease constantly present within a population, at a usual level of prevalence and in a stable state. An epidemic can turn into an endemic in one or both of the following cases: (i) loss of virulence of the pathogen; (ii) gradual elevation of specific antibodies in the affected population through repeated infections (which confers natural immunity) or regular vaccinations decided by public health authorities as a means of mitigation (antibody artificial induction). This decreases the population’s susceptibility to infection and the severity of infection in the individuals. This refers to a decrease in the pathogenicity of the infectious agent, which could make it either less infectious, or less lethal, or both [19], making the infection clinically stable and less apparent. Over time, the infectious agent usually mutates and circulates at lower, more manageable levels due to the occurrence of a variant more contagious, but less pathogenic. Then, a pandemic evolves into an endemic disease, the common example being influenza.

### 1.5. Overview of Epidemic/Endemic Transition: Example of Influenza

RNA viruses of influenza belong to the genus Orthomyxoviridae, and the first serious influenza pandemic occurred in 1918, infecting about one third of the entire world’s population until 1921 and killing patients in the range of 24.7–39.3 million [37]. Its dynamics has been well documented in some precise areas like New York City [38]. After its disappearance during the year 1921 in New Caledonia, the influenza virus mutated, resulting in descendant strains still circulating and infecting millions of patients and killing globally between 294,000 and 518,000 deaths every winter [39,40]. Influenza has become a sporadic disease, that is, a disease with epidemics occurring when a new virus strain appears into the population causing an antigenic drift [41], and between these epidemics, the virus continues to circulate between individuals in an endemic fashion, causing an infection to become clinically less apparent, making influenza a classic example of an endemic disease.

### 1.6. Organization of the Article

In the following, we introduce in Section 2 the criteria used to define the breakdown of stationarity of the random variable equal to the daily new cases of a contagious disease. Section 3 presents the results of an application concerning the COVID-19 outbreak. These results are discussed in Section 4, followed by some perspectives in Section 5 devoted to Conclusion.

## 2. Materials and Methods

### 2.1. Data Description

We considered COVID-19 daily empirical cases data in Japan, Nigeria, Cameroon, France, USA, and India. We chose countries in which either the level of economy (more or less developed) or the quality of detection (by more or less systematic PCR) or the vaccination policy (more or less generalized) or the dynamics of appearance of variants (more or less rapid) were different in order to obtain a representative sample of the different possible histories of the disease. Three countries are developed countries while others are developing countries. Three countries are among the seven most populated countries in the world, while others have between 30 and 70 million inhabitants. Two variants of SARS-CoV-2 were originated from India (Delta) and USA (Epsilon), which makes data sets interesting to gain insight in the dynamics of COVID-19 outbreak. 

We used the daily case count to analyze the differences in disease spread and peaks among these countries. For all the countries considered, daily numbers of confirmed cases, deaths, and full vaccinated data were extracted from public databases Worldometer [35] and Our World in Data [36] from January 2020 to July 2022.

**Figure 2:**
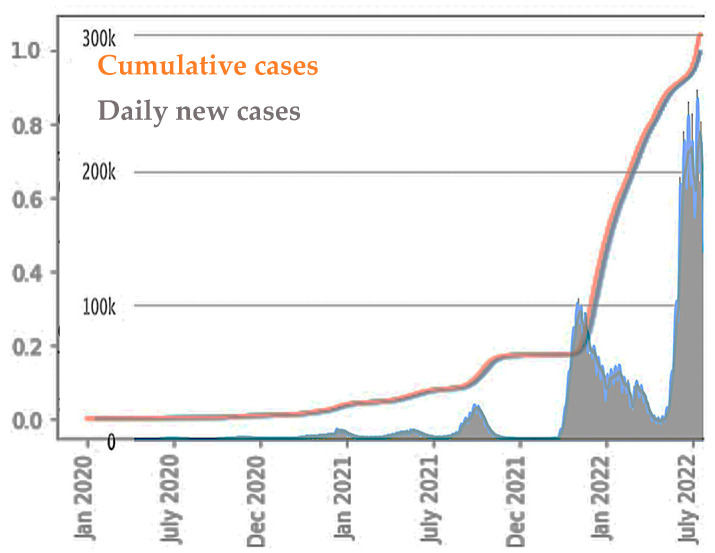
COVID-19 outbreak in Japan with Cumulative (resp. Daily new) cases in grey with a 7-day moving average in orange (resp. in grey with a 7-day moving average in light blue) (after [35]).

Figure 2 shows the real time of daily new and cumulative cases of COVID-19 for Japan, showing that since the initial stage of the epidemic, there were obvious differences between epidemic peaks. In this regard, we provide some explanations and insight to describe the observed phenomena in our analysis.

### 2.2. Stationarity Breakdown Criteria

The transition between the stationary endemic state of a contagious disease and an epidemic wave is studied by calculating three parameters in a moving window around the frontier on which we suspect that this transition occurred. These three parameters are the coefficient of variation, the entropy of the empirical distribution of the new cases of the disease daily observed, considered as random variable *N*, and the ratio between the absolute values of the dominant and subdominant eigenvalues of the transition matrix ruling the growth of *N*.

#### 2.2.1. Coefficient of Variation (CV)

The coefficient of variation of a random integer variable *N* valued in {*n*_1_, …, *n_d_*} is defined as the ratio of the standard deviation σ(*N*) to the mean *E*(*N*) of the empirical distribution of *N*, i.e., the set of weights *p_i_* = Proba({*N* = *n_i_*}*)* of the histogram:

(1) $$p_{i}=\frac{{Card(\{N=n}_{i}\})}{d}.$$

Then, the classical formulas for the first moments (expectation *E*(*N*) and standard deviation *σ*(*N*)) and the coefficient of variation CV of the empirical distribution {*p_i_*}*_i_*_=1,*d*_ are

(2) $$E\left(N\right)=\sum_{i=1}^{d}{N_{i}\;p}_{i},\;E\left(N^{2}\right)=\sum_{i=1}^{d}{{N_{i}}^{2}p}_{i},\;{\sigma (N)=[E\left(N^{2}\right)-{E\left(N\right)}^{2}]}^{\frac{1}{2}}\;,\;CV=\frac{\sigma \left(N\right)}{E\left(N\right)}\;.$$

#### 2.2.2. Empirical Entropy

The entropy E of the empirical distribution {*p_i_*}*_i_*_=1,*d*_ is defined as follows:


(3) $$E=-\sum_{i=1}^{d}p_{i}\log p_{i}.$$

The entropy E is maximal, equal to Log(*d*), when the empirical distribution is uniform, i.e., when all is equals 1/*d*, and E is minimal, equals 0, when only one *p_i_* equals 1.

#### 2.2.3. Spectral Subdominant/Dominant Ratio 

The Demongeot–Magal discrete equation of infectious dynamics is defined in [42]:

*S*(*t*) = *S*(0) − ∑*_i_*_=1,*t*−1_
*N*(*i*), (4)

where *S*(*t*) and *N*(*t*) are the numbers of susceptibles and infectious cases at day *t*. The transition matrix satisfies the Fröbenius theory; then, it has in its spectrum a real positive dominant eigenvalue λ_1_ and two complex conjugates as subdominant eigenvalues of absolute value $\left|\lambda_{2}\right|.$ Then, the spectral subdominant/dominant ratio *R* is defined as follows:

(5) $$R=\left|\lambda_{2}\right|/\lambda_{1}.$$

#### 2.2.4. Skewness

The skewness (*Skew*) of the empirical distribution {*p_i_*}*_i_*_=1,*d*_ of the random variable *N* is defined as its third standardized moment:


(6) $$\mathrm{Skew}=\sum_{i=1}^{d}{{\left(\frac{N_{i}-E(N)}{\sigma }\right)}^{3}\;p}_{i}.$$

#### 2.2.5. Kurtosis

The kurtosis (*Kurt*) of the empirical distribution {*p_i_*}*_i_*_=1,*d*_ of the random variable *N* is defined as its fourth standardized moment:


(7) $$\mathrm{Kurt}=\sum_{i=1}^{d}{{\left(\frac{N_{i}-E(N)}{\sigma }\right)}^{4}\;p}_{i}.$$

#### 2.2.6. Index of Dispersion

The index of dispersion (*ID*) is defined by the following formula:

*ID* = *σ*^2^(*N*)/*E*(*N*). (8)

*ID* equals 0 for a constant random variable *N* and 1 for a Poisson variable.

#### 2.2.7. Normality Index

The normality index *KStest* is defined as the fitting criterion of the Kolmogorov–Smirnov test of adequation to the normal distribution, with *E*(*N*) and *σ*(*N*) as, respectively, expectation and standard deviation of the empirical distribution of *N*.

### 2.3. Principal Component Analysis

The principal component analysis (PCA) is an exploratory data analysis technique which uses real data, for example, *q* variables for each individual of a population of size *n* (e.g., the observed COVID-19 new cases and deaths in the French population) [42,43,44]. Let us consider the *q n*-dimensional vectors $y_{j}$ made from these observations and calculate the combinations of the *y_j_*’s, which are orthogonal and have a variance decreasing with *i*. They constitute a matrix denoted as *Y* and defined as follows:

(9) $$\;\forall i=1,n,\;\sum_{j=1}^{q}y_{ji}a_{ji}=Ya_{i}\;or<y_{i},a_{i}>=\;Ya_{i}$$

$$and\;var\left(Ya_{i}\right)={a_{i}}^{T}Za_{i},$$

$$\mathrm{with}\;var\left(Ya_{1}\right)\geq var\left(Ya_{2}\right)\geq \ldots \geq var\left(Ya_{n}\right),$$

where $a_{i}$ is a vector commonly called the *i*^th^ eigenvector and *Z* is the covariance matrix associated with the real data. The *n* linear combinations $Ya_{i}$ are called principal components (PCs) and the elements of the eigenvectors $a_{j}$ are called PCs scores, which are values each among the *n* individuals score on PCs [43]. The first principal component $Ya_{1}$ offers the most information in the principal component analysis. 

### 2.4. Construction of a Score

In practice, the prediction power of each of the breakdown parameters is different from the others and can be measured in a retro-analysis by calculating the regression coefficients between the daily new cases *N*(*j*) observed at day *j* and the parameters calculated on a temporal moving window of two weeks ending on day *j*. We can then either retain the parameter with the greatest predictive power or define a breakdown score equal, in a multiple polynomial regression of the daily number of cases observed on the break parameters, to the combination of parameters producing the minimum error. A way to obtain this score is also to use the first principal component of principal component analysis (PCA), which explains, in general, a sufficient percentage of the variance of the new case empirical distribution.

### 2.5. Choice of the Countries

The choice of the studied countries has been guided by the search on three continents (Africa, Asia and Europe) of countries presenting complementary profiles to be compared in terms of values of mean Temperature (T), Elevation (E), Density (D), Age Median (M), *R_0_*, date of start and exponential slope of the first and second waves of new cases of COVID-19, and percentage of the GDP dedicated to health expenditure. These countries are selected as follows: for Africa, Cameroon and Nigeria; for Asia, Japan and India; for Europe, France and UK; and for North America, USA. Values of mean Temperature (T), Elevation (E), Density (D), Age Median (M), *R_0_*, date of start and exponential slope of the first and second waves of new cases of COVID-19, and percentage of the GDP dedicated to health expenditure are applied (cf. Table A1 in Appendix A).

## 3. Results

### 3.1. Indicators of Transition

#### 3.1.1. Coefficient of Variation (CV) during COVID-19 Outbreak

**Figure 3:**
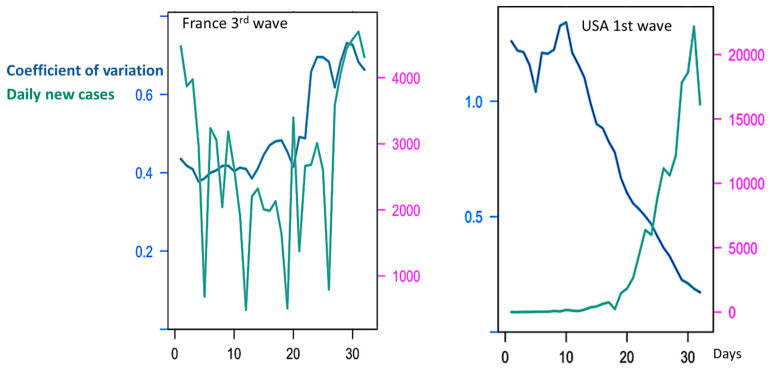
During France third (**left**) and USA first (**right**) endemic/epidemic transitions, the co-evolution of the Coefficient of Variation *CV* and Daily new cases. The x-axis represents time in days and the y-axes the Coefficient of Variation (in blue) and the Daily New Cases (in green).

CV alone is not a reliable predictor of epidemic waves due to varying trends among countries and waves. Figure 3 shows such variation of the coefficient of variation at the frontier between endemic and epidemic stages, but the sense of this variation varies largely between the waves in the same country and between countries. For example, CV decreases during first endemic/epidemic transition in the USA and India, but though in France it also decreases before the third wave, it increases during the fourth one (Figure 3 and Table 1).

#### 3.1.2. Empirical Entropy in COVID-19 Outbreak

**Figure 4:**
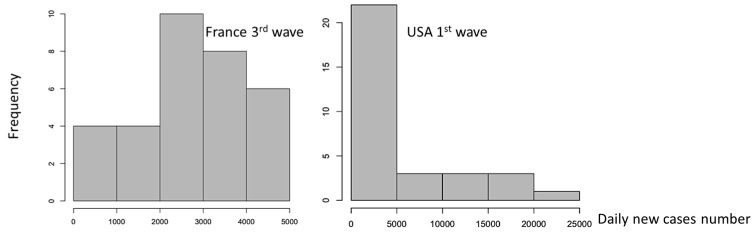
The empirical distributions of daily new cases for France 3rd wave and USA 1st wave.

**Figure 5:**
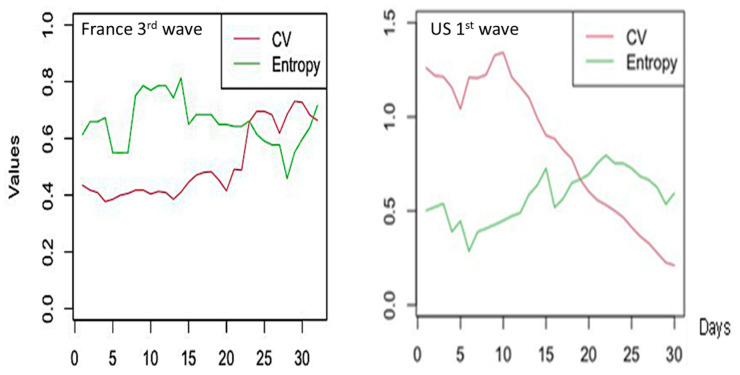
Co-evolution of CV and Entropy during France third (**left**) and USA first (**right**) waves.

Entropy is calculated for empirical distribution of daily new cases (Figure 4) and Figure 5 shows that Entropy alone is not a good predictor of new cases waves for France.

At the start of the first wave in the USA (Figure 5), we calculate the entropy E the value of which is equal to

$$-\sum_{i=1}^{6}p_{i}\log p_{i}=p_{1}\log p_{1}+p_{2}\log p_{2}+p_{3}\log p_{3}+p_{4}\log p_{4}+p_{5}\log p_{5}+p_{6}\log p_{6}=0.686.$$

#### 3.1.3. Spectral Dominant/Subdominant Ratio in COVID-19 Outbreak

*R* alone cannot represent a reliable endemic–epidemic transition predictor. In Table 2, we see that the values of the spectral ratio *R* increase during epidemic phases in France and Japan, but differences are very small and not significant.

### 3.2. Forecasting in COVID-19 Outbreak with a Reliable Score

Because the three first possible indicators of the endemic–epidemic transition have no prediction power, we keep the breakdown parameters calculated from the empirical distribution of the daily new cases, namely the coefficient of variation, the entropy, the third and fourth standardized moments (skewness and kurtosis), the uniformity index and normality index, all calculated on same moving window respecting the following rules:

- Choice of the same length of moving window as for the CV calculation (14 days);
- Use of the same time step as for moving the window (1 day);
- Movement of the window from the start to the end of the COVID-19 outbreak observed between January 2020 and July 2022.

**Figure 6:**
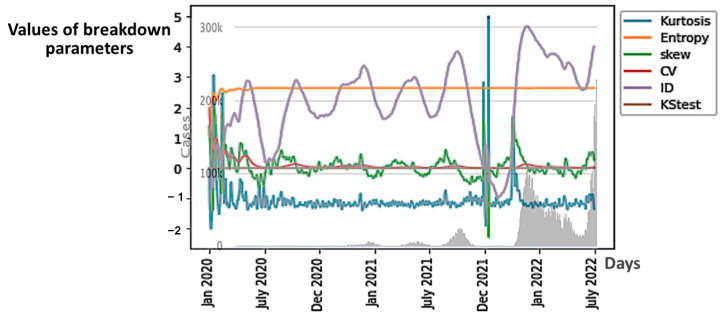
Breakdown Parameters and New Cases (in grey) in Japan during COVID-19 Outbreak.

**Figure 7:**
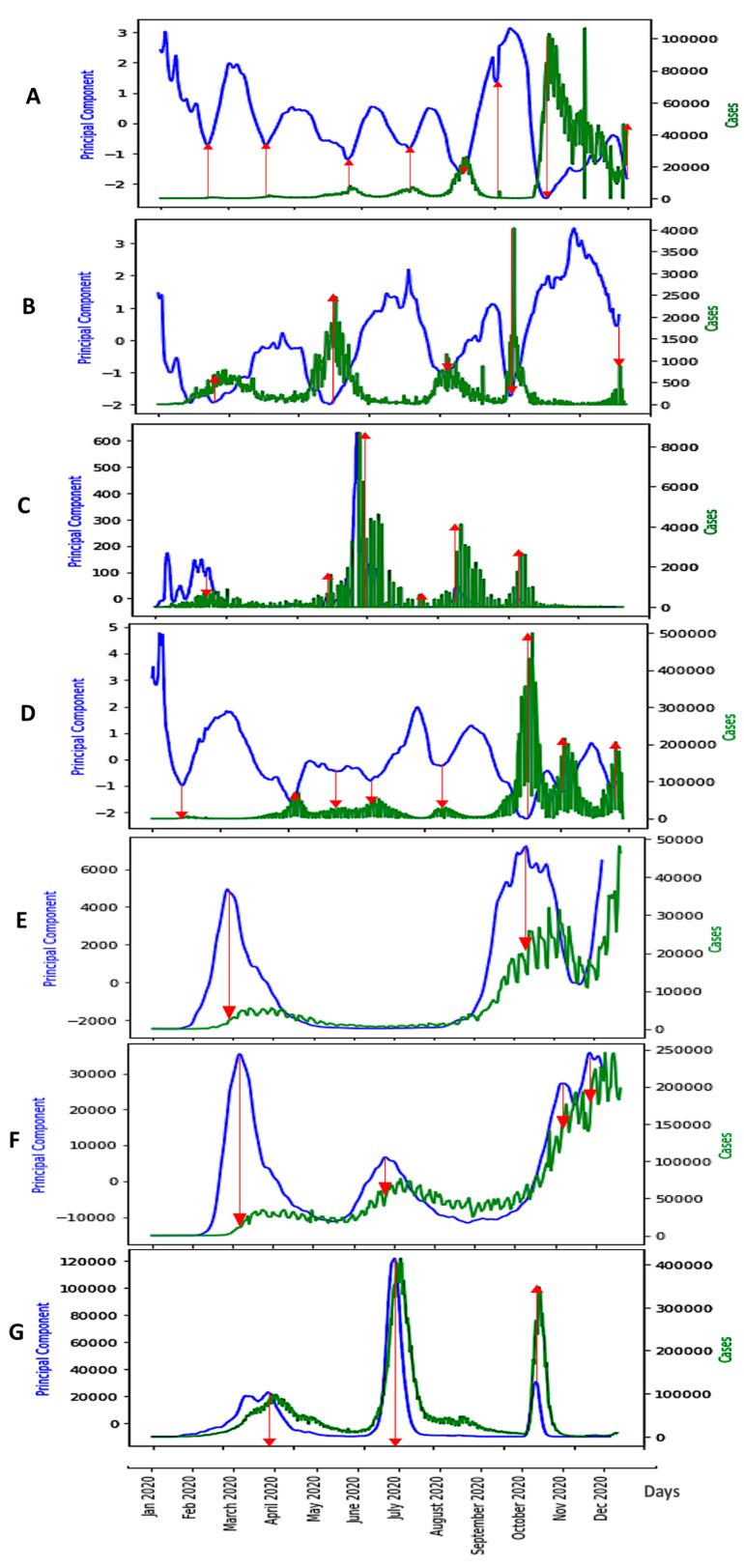
First Principal Component (blue) as predictor of COVID-19 Daily new case waves (green) in various countries: Japan (**A**), Nigeria (**B**), Cameroon (**C**), France (**D**), UK (**E**), USA (**F**) and India (**G**). The x-axis represents the time in days and the y-axis the PCA principal component. The red arrows correspond to local maxima of the first principal component.

In Figure 6, we can observe the evolution of all the six breakdown parameters in Japan, and in Figure 7A, we can observe that of only the first component of principal component analysis (PCA) performed with these parameters, which summarizes their predictive power globally. We can conclude that among the breakdown parameters, the only good predictor for epidemic waves is the first PCA component because its variations anticipate epidemic peaks.

In Figure 7, we can observe the evolution of only the first component of the PCA in seven different countries: Japan, Nigeria, Cameroon, France, UK, USA and India. We eliminated entropy and empirical moments because they have a restricted predictive power and the *ID* index because its predictive power is about same as that of PCA. We can observe that the minima of the PCA curves (blue) approximately correspond to the peaks of new cases curves (green) for the first four countries, but when endemic periods are long, PCA peaks are better predictors. It is the case for the UK, the USA and India, which contrasts with expectations.

## 4. Discussion

### 4.1. The ID Index as Predictor

**Figure 8:**
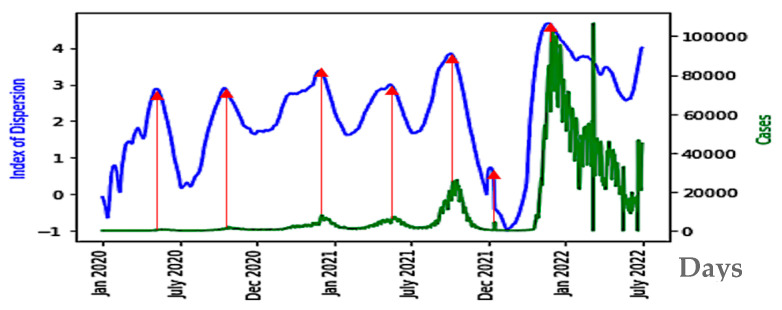
*ID* index (in blue) as predictor of the epidemic waves for Japan COVID-19 outbreak, with Daily new cases superimposed (in green). The x-axis represents the time in days. The red arrows correspond to local maxima of the first principal component.

The minima of the first PCA component curves correspond to the maxima of the *ID* index curves. Hence, *ID* index can be also a good predictor of COVID-19 epidemic peaks (Figure 8). 

In the case of Japan, the precision of the forecasting character of both the first PCA principal component *PCA1* and of the *ID* index can be easily explained by the fact that *ID* index often has the main weight in the linear combination expressing *PCA1* on the breakdown coefficients, as calculated for the first moving window in Japan during early January 2020, where the breaking coefficients are calculated for the first moving windows of two weeks in Table 3: *PCA1* = 8.86760799 10^-2^ *Kurt* + 1.73156383 10^-2^ *E* + 1.25157924 10^-2^ *Skew* + 2.49657969 10^-2^ *CV* + 9.95518350 10^-1^ *ID* + 1.05368220 10^-5^ *KS*. 

The values of the breakdown variable *ID* remain small during the COVID-19 evolution, but their relative variations ∆*ID*(*i*) = [*ID*(*i* + 1) − *ID*(*i*)]/*ID*(*i*) are important (Table 3), which explains the relatively important weight of *ID* in *PCA1*. The minima of *PCA1* and maxima of *ID* are systematically preceding the epidemic peaks (except for India), and the change in nature in the empirical distribution (the loss of stationarity) of the new cases is easily understandable. The index of dispersion *ID* is indeed the logarithm of the ratio between second and first moments of the empirical distribution of new cases and its variations reflect the loss of stationarity before an exponential growth of the new cases, which is the main characteristics of the early dynamics of an epidemic peak. We observe the same predictive behavior for the breakdown parameters and PCA1, calculated from death data.

### 4.2. The Influence of Vaccination on the Daily New Cases and Deaths Curves

**Figure 9:**
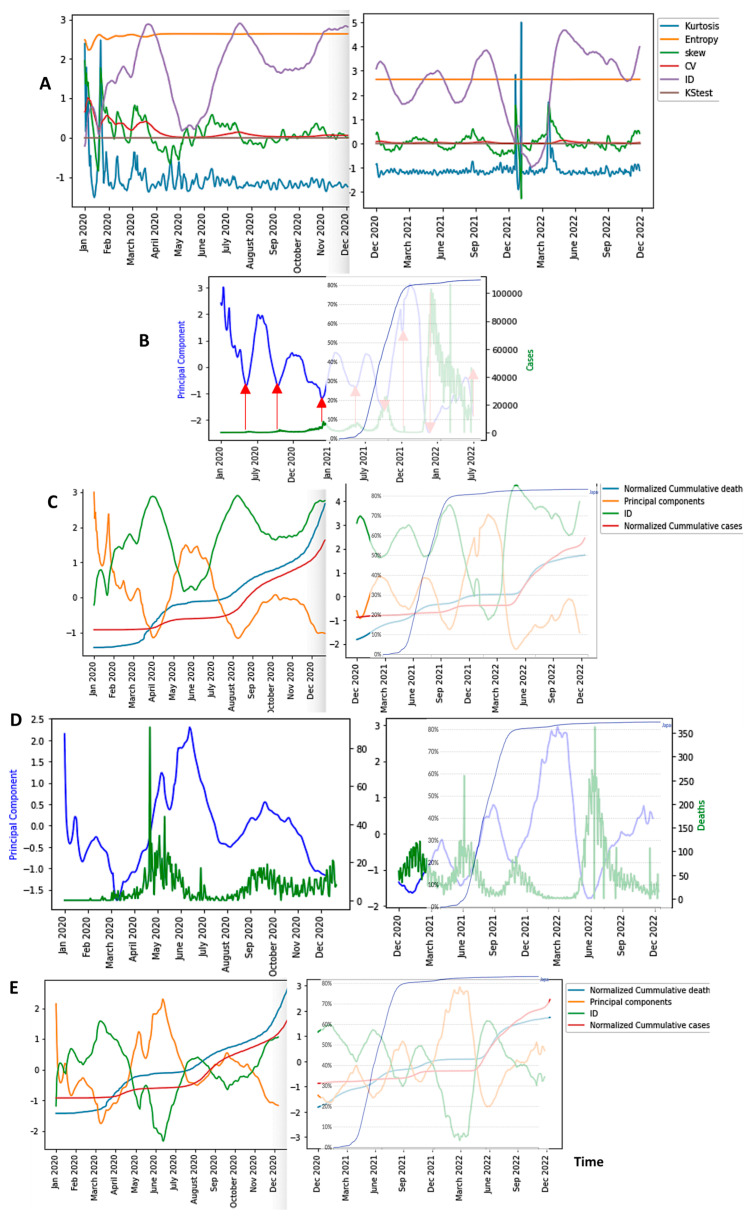
(**A**) Breakdown parameters of new cases before (**left**) and after (**right**) vaccination during Japan COVID-19 outbreak; (**B**) Influence of vaccination on waves, with PCA1 (in blue) and new cases (in green) before (**left**) and after (**right**) vaccination with percentage of fully vaccinated superimposed (in light blue); (**C**) PCA1 and ID for new cases before and after vaccination (fully vaccinated superimposed); (**D**) PCA1 for deaths before (**left**) and after (**right**) vaccination (fully vaccinated superimposed); (**E**) PCA1 and ID for deaths before (**left**) and after (**right**) vaccination (fully vaccinated superimposed). The x-axis represents the time (in months). The red arrows correspond to local maxima of the first principal component.

**Figure 10:**
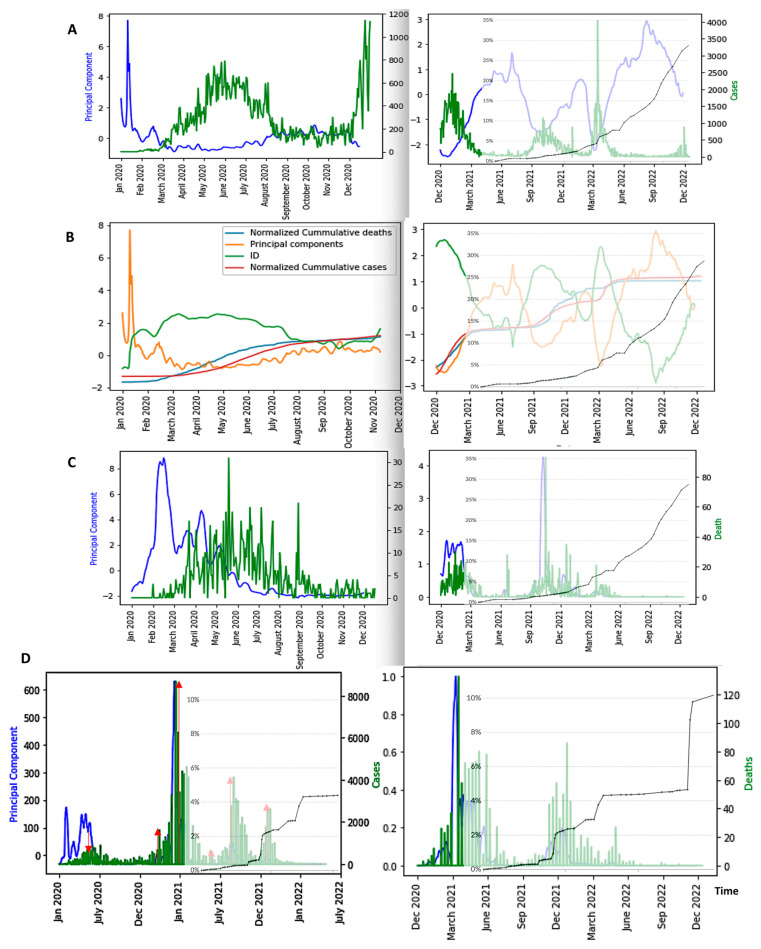
(**A**) Influence of vaccination on waves of Nigeria COVID-19 outbreak, with PCA1 (in blue) and daily new cases (in green) before (**left**) and after (**right**) vaccination with percentage of fully vaccinated people superimposed (in black); (**B**) PCA1 and ID for new cases and deaths before and after vaccination (percentage of fully vaccinated superimposed); (**C**) PCA1 for deaths before (**left**) and after (**right**) vaccination (fully vaccinated superimposed); (**D**) same as (**A**) and (**C**) for Cameroon with new cases (**left**) and deaths (**right**) superimposed (in green) with fully vaccinated superimposed (in black). The x-axis represents the time (in months). The red arrows correspond to local maxima of the first principal component.

**Figure 11:**
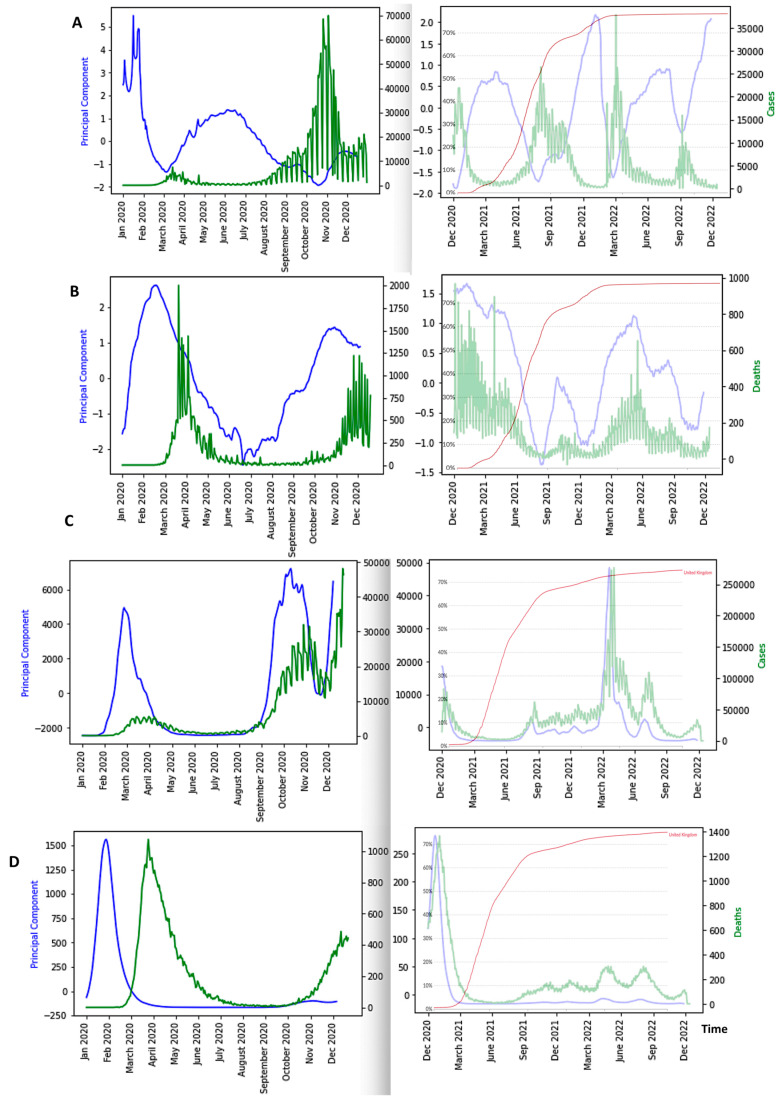
(**A**) Influence of vaccination on waves of France COVID-19 outbreak, with daily new cases superimposed (in green) before (**left**) and after (**right**) vaccination with percentage of fully vaccinated people superimposed (in red); (**B**) same for deaths before and after vaccination; (**C**) same as (**A**) for the United Kingdom; (**D**) same as (**C**) for the US. The x-axis represents the time (in months). The red arrows correspond to local maxima of the first principal component.

Figure 9, Figure 10 and Figure 11 show the influence of vaccinations on new cases and deaths curves.

**Figure 12:**
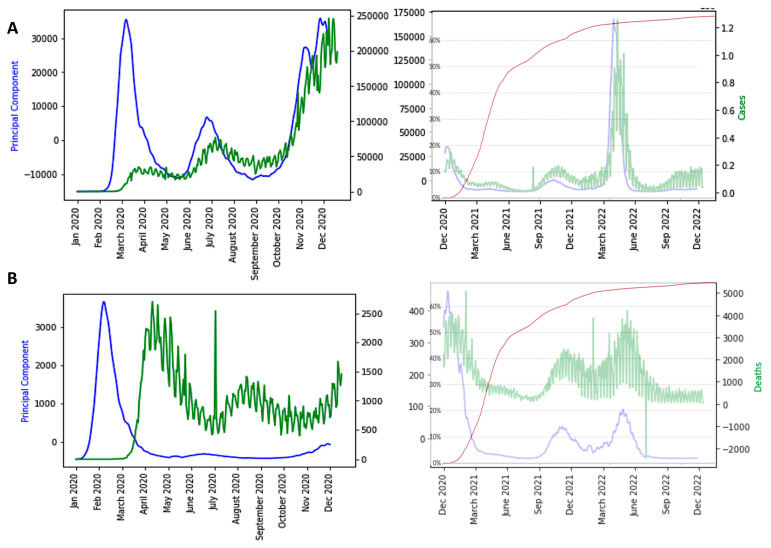

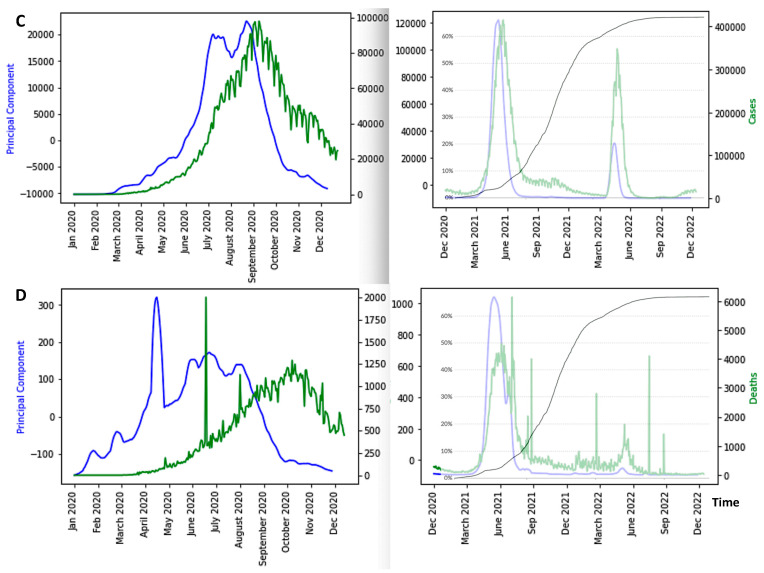
(**A**) Influence of vaccination on waves USA COVID-19 outbreak, with daily new cases superimposed (in green) before (**left**) and after (**right**) vaccination with percentage of fully vaccinated people superimposed (in red); (**B**) same for deaths before and after vaccination; (**C**) same as (**A**) for India; (**D**) same as (**C**) for India. The x-axis represents the time (in months). The red arrows correspond to local maxima of the first principal component.

We see in Figure 9, Figure 10, Figure 11 and Figure 12 the following three main features:

- The first PCA component (PCA1) anticipates systematically the new case and death waves, the latter ones occurring some weeks (between two and four) after the new case waves;
- ID waves occur in opposition of phases with PCA1, but also predicts the new case and death waves well;
- This anticipation remains true after vaccination, except for the end of the vaccination campaign which shows the beginning of a decorrelation between PCA1 and new case last waves.

These features have to be confirmed in future works. Suggested directions could be:

(i) to develop a parametric model that considers all the variables and parameters necessary for modeling the endemic/epidemic transition;
(ii) to test the predictive power of the breakdown parameters used in the present article on other variables linked to the COVID-19 outbreak as the number of deaths, hospitalizations and ICU sojourns;
(iii) to examine past outbreaks concerning other infectious diseases like Influenza H1N1 in 1977 or Ebola in Sierra Leone during the years of 2014 and 2015 and in Democratic Republic of Congo in 1995, and test for these infectious diseases the retro-predictive power of PCA1.

## 5. Conclusions

We examined the predictive power of seven parameters related to the empirical distribution of new COVID-19 cases in six countries (Japan, Nigeria, Cameroon, France, USA, and India), which constitutes an improvement of our previous work on COVID-19 outbreak in [44,45,46,47] using different approaches. Only six parameters showed an ability to predict epidemic peaks, all being related to the empirical distribution of new cases: kurtosis, entropy, skewness, coefficient of variation, index of dispersion and the fitting criterion of the Kolmogorov–Smirnov normal adequation test. The calculation of the first component of principal component analysis (PCA) based on these six parameters showed that its principal component *PCA1* has a good forecasting power in all the above-mentioned countries, except the USA and India, whose endemic phases showed only weak variations of the moments of the empirical distribution of the Daily new cases. Hence, for the USA and India, a minimum of the *ID* variable was impossible to individualize inside the endemic background noise. The future efforts in the direction of this research are vital for a future pandemic or emerging infectious disease preparation, because we believe that the research presented in this article could be relevant for new infectious case forecasting in order to deploy proper intervention and resources (as vaccination policies [48]) to fight the epidemic spread and, in a way, guide policy-making for public health.

## Figures and Tables

**Table 1:** *CV* values for USA (1st and 4th wave) and India (1st, 2nd, 3rd, 4th waves) during epidemic waves of COVID-19 (after [23]).

United states *CV* values start of the pandemic 27/02/2020 - 1.259872 12/03/2020 - 0.9012443 26/03/2020 - 0.2257 fourth wave 01/06/2021 - 0.373792 15/06/2021 - 0.4426645 01/07/2021 - 0.5956981 India *CV* values start of the pandemic 02/03/2020 - 0.9011401 16/03/2020 - 0.6828228 01/04/2020 - 0.4409121 Second wave 23/01/2021 - 0.1967528 06/02/2021 - 0.1313073 20/02/2021 - 0.1390496 Third wave 01/11/2021 - 0.0856899 15/11/2021 - 0.1311902 01/12/2021 - 0.4360979 Fourth wave 01/05/2022 - 0.1407748 15/05/2022 - 0.1719229 02/06/2022 - 0.3735433	United states *CV* values start of the pandemic 28/02/2020 - 1.219726 13/03/2020 - 0.8833425 27/03/2020 - 0.2107585 fourth wave 02/06/2021 - 0.3787229 16/06/2021 - 0.462615 02/07/2021 - 0.5756484 India *CV* values start of the pandemic 03/03/2020 - 0.8479051 17/03/2020 - 0.7268929 02/04/2020 - 0.4300673 Second wave 24/01/2021 - 0.1920922 07/02/2021 - 0.1423886 21/02/2021 - 0.1463374 Third wave 02/11/2021 - 0.1042972 16/11/2021 - 0.1506754 02/12/2021 - 0.4391623 Fourth wave 02/05/2022 - 0.1613438 16/05/2022 - 0.1773734 03/06/2022 - 0.3925422	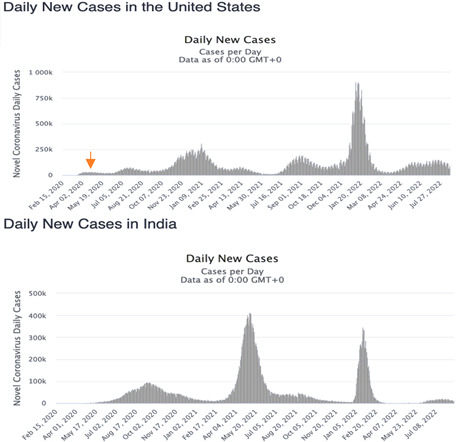

**Table 2:** Absolute value of dominant and first subdominant eigenvalues, and spectral ratio *R* = $\left|\lambda_{2}\right|/\lambda_{1}$.

**France**	$\lambda_{1}$	$\left|\lambda_{2}\right|$	$R=\left|\lambda_{2}\right|/\lambda_{1}$
Period 1: Epidemic phase 27 February–17 May 2020	1.028886	1.015612	0.987106
Period 2: Endemic phase 17 May–17 July 2020	1.002432	1.002580	1.00015
Period 3: Epidemic phase 15 September–26 November 2020	1.003880	0.981878	0.978083
Period 4: Endemic phase 26 November–20 December 2020	1.019847	1.021709	1.00183
Period 5: Epidemic phase 20 December–25 February 2021	1.005828	0.991934	0.986186
Japan	$\lambda_{1}$	$\left|\lambda_{2}\right|$	$\left|\lambda_{2}\right|/\lambda_{1}$
Period 1: Epidemic phase 20 February–27 May 2020	1.028575	1.022287	0.993887
Period 2: Endemic phase 27 May–13 June 2020	1.002512	0.773729	0.771790
Period 3: Epidemic phase 13 June–10 September 2020	1.020337	1.014091	0.993879
Period 4: Endemic phase 10 September–18 October 2020	1.005970	0.989558	0.983686
Period 5: Epidemic phase 18 October–5 December 2020	1.039391	1.040991	1.001539

**Table 3:** Values of the breakdown coefficients during the first two weeks moving windows *W*(*i*) (*i* = 0 to 4) for Japan during early January 2020.

*i*	*Kurtosis*	*Entropy*	*Skew*	*CV*	*ID*	*KStest*	∆*ID*
0	−0.06	1.1	1.39	1.99	−0.07	0.00092	0.57
1	−1.1	1.39	0.95	1.64	−0.11	0.00092	0.40
2	−1.64	1.61	0.60	1.39	−0.16	0.00092	0.32
3	−1.92	1.79	0.29	1.20	−0.21	0.00092	0.28
4	−2.0	1.95	0	1.04	−0.27	0.00092	

## Data Availability

All data used in the present article come from public data bases cited in References.

## Appendix A

**Table A1:** Values of mean Temperature (T), Elevation (E), Density (D), Age Median (M), *R_0_*, date of start and exponential slope of the first and second waves of new cases of COVID-19, and percentage of the GDP dedicated to health expenditure.

Country	T (°C)	E (m)	D (h/km^2^)	AM (years)	1st wave R_0_ /Date start	1st Wave Exponential Slope	2nd Wave R_0_/Date Start	2nd Wave Exponential Slope	GDP % Health 2020
**Africa**									
Algeria	22.5	800	18	28.1	2.19/20–03	0.1594	0.86/07–04	0.0316	6.32
**Cameroon**	**31**	**667**	**56**	**17.7**	**2.56/03–04**	**0.0338**	**1.64/07–07**	**0.0085**	**3.77**
Djibouti	28.0	430	47	23.9	2.31/07–04	0.489	2.47/07–05	0.5	2.01
Guinea	25.7	472	50	18.9	1.5/04–04	0.0744	1.36/28–06	0.01	4.04
Mauritius	22.4	209	620	35.3	5.4/25–03	0.02	9.32/02–06	0.03	5.83
Morocco	17.1	909	80	29.3	2.05/21–03	0.1161	0.84/21–05	0.0687	5.31
**Nigeria**	**26.8**	**30**	**229**	**18.4**	**1.96/29–03**	**0.0672**	**1.25/02–05**	**0.0258**	**3.38**
Senegal	27.85	69	82	18.8	2.02/12–04	0.1003	1.24/17–05	0.0238	3.98
South Africa	22.5	1034	49.1	27.6	2.48/07–03	0.257	1.15/06–05	0.0303	6.25
Sudan	26.9	568	22	19.9	1.97/20–04	0.0193	1.19/09–06	0.0407	4.51
Tunisia	19.2	246	186	31.6	1.34/15–03	0.01	2.64/24–06	0.142	7.29
**Asia**									
Bangladesh	25.0	85	1175	26.7	3.67/05–04	0.0399	0.92/01–08	0.01	2.34
**India**	**27.4**	**160**	**470**	**32.4**	**2.43/22–04**	**0.0331**	**0.91/15–11**	**0.01**	**3.54**
Iran	24	1305	51	30.3	3.61/04–03	0.2641	1/01–05	0.0438	8.66
Iraq	14.03	312	90	20.0	1.81/14–03	0.1184	0.96/18–07	0.0410	5.1
Israel	19.2	508	417	29.9	2.86/05–03	0.005	1.33/19–05	0.0339	7.52
**Japan**	**11.15**	**438**	**333**	**47.3**	**1.91/25–02**	**0.0872**	**1.21/21–06**	**0.0260**	**10.95**
Pakistan	20.20	900	274	23.8	1.90/15–03	0.1301	1.02/01–09	0.0113	3.20
Turkey	11.1	1132	106	30.9	4.32/11–03	0.0120	0.81/02–06	0.0473	4.12
**Europa**									
Albania	11.4	708	100	32.9	1.61/23–03	0.0309	0.99/18–05	0.0825	5.26
Austria	6.35	910	106	44.0	2.93/08–03	0.2825	1.05/07–06	0.0545	10.33
Belgium	9.55	181	378	41.4	8.28/06–03	0.1963	0.88/16–06	0.0257	10.32
Bosnia/Her.	9.85	500	69	42.1	1.70/21–03	0.1671	0.97/05–06	0.0667	8.90
Bulgaria	10.55	472	64	42.7	1.97/19–03	0.0927	0.78/13–04	0.0049	7.35
Croatia	10.9	331	187	43	3.95/18–03	0.093	0.72/12–06	0.01	6.83
Denmark	7.5	34	349	42.2	1.60/05–03	0.01	0.90/07–07	0.0539	10.07
**France**	**10.7**	**375**	**123**	**41.4**	**2.68/29–02**	**0.2898**	**1/24–06**	**0.01**	**11.26**
Finland	1.7	134	16	42.5	1.66/12–03	0.0711	1.04/26–07	0.0891	9.02
Greece	15.4	498	210	44.5	1.72/11–03	0.0759	1.05/24–06	0.01	7.72
Georgia	5.8	1432	54	38.1	2.19/30–03	0.0346	0.76/02–06	0.1471	7.11
Germany	8.5	263	233	47.1	2.84/29–02	0.2624	0.98/10–06	0.005	11.43
Hungary	9.75	143	272	42.3	2.25/21–03	0.0586	0.77/01–07	0.01	6.7
Luxemburg	8.65	325	237	39.3	1.99/16–03	0.4841	0.83/01–06	0.0271	5.29
Malta	19.2	1	1567	41.8	4.46/23–03	0.0712	1.29/19–04	0.0536	8.96
Moldova	9.45	139	79	36.7	2.03/22–03	0.1716	0.83/31–04	0.0217	6.6
N Macedonia	9.8	741	81	37.9	1.84/21–03	0.0858	0.87/03–05	0.028	6.58
Netherlands	9.25	30	421	42.6	2.4/05–03	0.2485	0.92/07–07	0.0002	9.97
Norway	1.5	460	17	39.2	2.4/09–03	0.2716	1.14/19–07	0.1725	10.05
Poland	7.85	173	123	40.7	2.17/14–03	0.1562	0.99/05–04	0.0094	6.33
Romania	8.8	414	81	41.1	2.26/14–03	0.1596	0.91/31–05	0.0498	5.56
Serbia	10.55	473	89	42.6	2.13/19–03	0.1919	0.79/01–06	0.0123	8.54
Slovenia	8.9	492	266	44.5	1.78/17–03	0.1301	1.08/12–06	0.01	8.3
Spain	13.3	660	93	42.7	3.85/25–02	0.335	1.16/29–06	0.0846	8.98
Sweden	2.1	320	23	41.2	2.10/05–03	0.2572	1.05/24–05	0.0768	10.9
Switzerland	5.5	1350	208	42.4	2.86/04–03	0.2388	0.95/08–06	0.0664	11.88
**UK**	**8.45**	**162**	**280**	**40.5**	**2.89/04–03**	**0.2223**	**1.25/02–07**	**0.0416**	**10**
Ukraine	8.3	175	70	40.6	2.16/24–03	0.1615	0.89/25–05	0.048	7.72
**North America**									
Canada	−5.35	487	4	42.2	2.95/10–03	0.2432	1.05/01–07	0.0153	10.79
Cuba	25.2	108	102	41.5	2.23/27–03	0.0706	1.30/17–05	0.0517	11.19
Dominican Republic	24.55	424	34	38.1	2.09/20–03	0.1403	1.10/01–06	0.0151	5.73
**USA**	**8.55**	**760**	**34**	**38.1**	**3.85/02–03**	**0.2882**	**0.99/07–06**	**0.0119**	**16.89**
